# Molecular-genetic approaches to species identif ication of platyhelminthes of the genus Ligophorus (Monogenea) parasitising f lathead mullet

**DOI:** 10.18699/VJGB-22-36

**Published:** 2022-05

**Authors:** E.A. Vodiasova, E.S. Chelebieva, O.V. Shikhat, D.M. Atopkin, E.V. Dmitrieva

**Affiliations:** A.O. Kovalevsky Institute of Biology of the Southern Seas of the Russian Academy of Sciences, Sevastopol, Russia; A.O. Kovalevsky Institute of Biology of the Southern Seas of the Russian Academy of Sciences, Sevastopol, Russia; A.O. Kovalevsky Institute of Biology of the Southern Seas of the Russian Academy of Sciences, Sevastopol, Russia; Federal Scientific Center of the East Asia Terrestrial Biodiversity, Far Eastern Branch of the Russian Academy of Sciences, Vladivostok, Russia; A.O. Kovalevsky Institute of Biology of the Southern Seas of the Russian Academy of Sciences, Sevastopol, Russia

**Keywords:** genotyping, allele-specific PCR, Monogenea, Ligophorus, Mugil cephalus, генотипирование, аллель-специфическая ПЦР, Monogenea, Ligophorus, Mugil cephalus

## Abstract

Mugil cephalus L., 1758 (flathead mullet) is a valuable commercial fish and a promising object of artificial breeding in the Black Sea and the Sea of Azov, and the study of its parasite fauna is important for fishery and mariculture. Monogeneans of the genus Ligophorus are common ectoparasites dwelling on the gills of mullets. Two representatives of this genus parasitise flathead mullet in the Azov-Black Sea region, namely Ligophorus mediterraneus Sarabeev, Balbuena et Euzet, 2005 and Ligophorus cephali Rubtsova, Balbuena, Sarabeev, Blasco- Costa et Euzet, 2006. Morphological identification of these species requires spending much time and a high level of experience in monogenean taxonomy. For quick and correct species identification of these parasites, we have developed a genotyping approach based on the polymerase chain reaction of allele-specific gene sites for various Monogenea species. A fragment of the 28S ribosomal gene, which includes conserved and variable sites, was chosen as a genetic marker. Three approaches were used as follows: amplified fragment length analysis, allelespecific PCR with endpoint detection and allele-specific real-time PCR using SYBR Green intercalating dye. The first approach was by obtaining PCR products of different lengths that were specific either to L. mediterraneus or to L. cephali. This approach was implemented due to the presence of several variable sites located at a distance from each other. The PCR mixture contained three primers: one forward and two reverse. The forward primer was complementary to the conserved site, which did not differ between species. Reverse primers were speciesspecific and, for each species, they were complementary to different DNA regions located 100 bp apart. As a result, L. mediterraneus was characterized by shorter amplicons than L. cephali. For the second and third approaches, a pair of primers was designed according to the following principle: the forward primer was complementary to both species, since it was selected for the conserved gene region. Reverse primers were species-specific and were designed for the 28S variable region. The two parasite species were distinguished by three-point mutations. Thus, one pair of primers was complementary to L. mediterraneus, the other, to L. cephali. The amplified fragment length analysis and the allele-specific real-time PCR demonstrated 100 % coincidence of genotyping results compared with Sanger sequencing. The developed genotyping protocols can be used not only to distinguish two species of Ligophorus from flathead mullet in ecological studies and veterinary practice but also for further development of similar approaches for other monogeneans, among which there are many pathogenic species.

## Abstract (ru)

Mugil cephalus L., 1758 (лобан) – ценная промысловая рыба и перспективный объект разведения в бассейнах Черного и Азовского морей. Изучение его паразитофауны крайне важно для рыбного промысла и марикультуры. Одними из массовых эктопаразитов, обитающих на жабрах кефалевых, являются моногенеи рода Ligophorus. На лобане в Азово-Черноморском регионе паразитируют два представителя этого рода: Ligophorus mediterraneus Sarabeev, Balbuena et Euzet, 2005 и Ligophorus cephali Rubtsova, Balbuena, Sarabeev, Blasco-Costa et Euzet, 2006. Морфологическое определение этих видов весьма трудоемко и требует высокого уровня квалификации. Для быстрого и точного различия двух названных видов паразитов нами разработан метод, основанный на ПЦР вариабельных участков рибосомного гена 28S. Данный ген широко используется для молекулярной таксономии рода Ligophorus. В настоящей работе применялись три подхода: анализ длины амплифицированных фрагментов, аллель-специфичная ПЦР с детекцией в конечной точке и аллель-специфичная ПЦР в реальном времени с использованием интеркалирующего красителя SYBR Green. Первый подход заключался в подборе праймеров для получения ПЦР-продуктов различной длины, которые были характерны для L. mediterraneus или L. cephali. Этот подход был осуществлен благодаря наличию нескольких вариабельных локусов, которые находятся на расстоянии друг от друга. ПЦР-смесь содержала три праймера: один прямой и два обратных. Прямой праймер был комплементарен консервативному участку, который не различался между видами. Обратные праймеры были видоспецифичны, при этом для каждого вида они были комплементарны различным участкам ДНК, которые расположены на удалении 100 п. н. друг от друга. В результате L. mediterraneus характеризовался более короткими ампликонами, чем L. cephali. Для второго и третьего подхода конструировалась пара праймеров по следующему принципу: прямой праймер был комплементарен обоим видам, так как подбирался к консервативному участку гена. Обратные праймеры были видоспецифичными и разрабатывались к вариабельному участку 28S. На этом участке два вида паразита различались тремя точечными мутациями. Таким образом, одна пара праймеров была комплементарна L. mediterraneus, вторая – L. cephali. Анализ длины амплифицированных фрагментов и аллель-специфичная ПЦР в реальном времени продемонстрировали 100 % совпадение результатов генотипирования при сравнении с морфологической идентификацией и секвенированием по Сэнгеру. Разработанные протоколы генотипирования могут быть использованы не только для различия обитающих на лобане двух видов Ligophorus при экологических исследованиях и в ветеринарной практике, но и для последующей разработки подобных методов для других моногеней, среди которых много патогенных видов.

## Introduction

Monogenea (Platyhelminthes: Monogenea) are parasites, mainly of fish, with a direct life cycle. Dozens of new taxa of these parasites are described each year. Their diversity has reached 5000 species (Vanhove, 2013), and many of them are of epizootic importance (Cribb et al., 2002; Bakke et al., 2007; Rubio-Godoy, 2007). The boundaries of most species are established based on morphological criteria, and for species identification, the shape and size of attachment disc structures are mainly used (Yamaguti, 1963; Gusev et al., 1985; Pugachev et al., 2009; Vignon, 2011; Strona et al., 2014; Kalafi et al., 2016). However, these structures have high intraspecific variability (Ergens, Gelnar, 1985; Caltran et al., 1995; Dmitrieva, Dimitrov, 2002; Olstad et al., 2009; Mladineo et al., 2013). The latter makes it very difficult to determine the species identity of monogeneans and raises the question of defining the framework of their intra- and interspecific variability. Appealing to real collection specimens to confirm the determination is often difficult due to accessing collections with type specimens. Comparison of organisms with many “similar” species from different areas based on brief descriptions and often inaccurate drawings does not always allow reliable species identification. As a result, an increase in the number of “false” and underestimation of “hidden” species taxa can lead to misunderstanding of the phylogeny, diversity and distribution of representatives of individual monogenean groups (Poisot et al., 2011), and sometimes to problems in determining the status of pathogenic species, as in the case of Gyrodactylus salaris and G. thymalli (Fromm et al., 2014; Mieszkowska et al., 2018).

Given the above, the development of approaches and methods allowing for the most accurate identification of monogenean species remains an urgent task, both in theoretical and practical terms. One of the promising directions in molecular genetic studies of parasites is the development of methods for genotyping species and local intraspecific groupings, both for biodiversity studies of individual taxa and for rapid diagnosis of species and their populations (Tokarev et al., 2015). Such works with relation to monogeneans are rare (Fromm et al., 2013, 2014; Mieszkowska et al., 2018). A few papers address the problems of DNA barcoding of monogenean species (Littlewood, 2008; Vanhove, 2013). Molecular studies on the genus Ligophorus Euzet et Suriano, 1977 are limited to a few studies, with 135 ribosomal nuclear DNA sequences deposited in the NCBI GenBank (as of 27.11.2021). The 18S, ITS1, 5.8S and 28S fragments were obtained for 12 species from the Mediterranean Sea and 2 species from the Black Sea (Blasco-Costa et al., 2012; Rodríguez-González et al., 2015). For two species off the coast of Brazil, 18S, ITS1, 5.8S and 28S were sequenced (Marchiori et al., 2015), and 18S, 28S and ITS1 fragments were obtained for 14 species from the Indian Ocean (Soo et al., 2015; Khang et al., 2016; Pakdee et al., 2019). Several studies (Blasco-Costa et al., 2012; Rodríguez-González et al., 2015; Khang et al., 2016) have compared morphological and genetic variability, showing a greater degree of congruence between phylogenetic reconstructions based on these data, suggesting that the use of ribosomal cluster sequences for genotyping species of this genus is promising.

The flathead mullet Mugil cephalus L., 1758 is a commercial fish of the Black and Azov Seas and a promising object of mariculture in the region; therefore, the study of its parasitofauna is critical not only from the scientific but also from the practical point of view. Monogeneans of the genus Ligophorus, which parasitise on the gills of mullets, are one of the ectoparasites for the flathead mullet. In the Azov- Black Sea region, L. mediterraneus Sarabeev, Balbuena et Euzet, 2005 and L. cephali Rubtsova, Balbuena, Sarabeev, Blasco-Costa et Euzet, 2006 have morphologically similar attachment structures (Dmitrieva et al., 2009a, b), which makes their identification difficult. At the same time, these species have a good level of genetic divergence based on the variability of 28S and ITS1 (Blasco-Costa et al., 2012). This divergence is due to the single nucleotide substitutions characteristic of L. cephali and L. mediterraneus. When assessing the infestation of these species in large samples of fish, e. g. in ecological or veterinary surveys, the use of morphological characters is problematic, and sequencing followed by molecular taxonomy is costly and time-consuming. In addition, up to eight Ligophorus species may parasite on one individual mullet (Dmitrieva et al., 2012; Soo et al., 2015). This situation is not unique and occurs for species of the same genus in many members of the family Dactylogyridae, which includes Ligophorus.

With the appearance of real-time PCR, alternative approaches for genotyping based on allele-specific PCR that allows rapid and reliable species identification have begun to develop. However, for members of the family Dactylogyridae, such approaches have not been used. Thus, this work aimed to develop an express methodology to distinguish two monogenic species L. cephali and L. mediterraneus parasitising on the proboscis in the Azov-Black Sea region based on 28S gene variability. Considering that there are many representatives of epizootic importance among Dactylogyridae, the development of inexpensive and straightforward methods for rapid genotyping of species of this taxon to distinguish between pathogenic and nonpathogenic species, including at the larval stage, is also relevant in a practical sense.

## Materials and methods

Sampling. The material for this work was 20 specimens of monogeneans of the genus Ligophorus collected from the gills of 3 individuals of Mugil cephalus in autumn 2019 in the Black Sea off the coast of Crimea, in Balaklava Bay. The worms were collected alive, a glycerol-gelatin preparation (Gusev, 1983) was prepared from part of an individual for species identification by the morphology of the attachment disc structures, and another part of the same monogenean was fixed in 96 % ethanol for molecular genetic studies.

Taxonomy identification. Species identification based on the shape and size of the haptoral structures and male copulatory organ of monogeneans using an Olympus CX41 microscope and phase-contrast optics at ×800–2,000 magnification according to the descriptions of Ligophorus species from Black Sea mullet (Dmitrieva et al., 2009a, b). Measurements and photographs were taken using CellSense digital image processing software.

DNA isolation and genetic analyses. The isolation was performed using a DNA-EXTRAN kit (Sintol Ltd., Russia). Each individual was incubated in 100 μL of lysis buffer (Sintol Ltd.) with 5 μL of proteinase K (Sintol Ltd.) and 1 μL of 2-mercaptoethanol at 56 °C for 3 hours. After lysis, samples were shaken for 20 s and further DNA extraction was performed according to the manufacturer’s recommendations. DNA elution was performed in 30 μL. The isolated DNA was stored at –20 °C.

For the molecular taxonomy of the species, the 28S ribosomal gene, which is used in the analysis of this genus, was chosen as a genetic marker (Blasco-Costa et al., 2012; Soo et al., 2015; Pakdee et al., 2018). The 28S gene fragment was amplified using primers U178 (5′-GCACCCGCT GAAYTTAAG-3′ ) and LSU1200R (5′-GCATAGTTCAC CATCTTTCGG-3′ ) (Littlewood et al., 2000; Lockyer et al., 2003) according to the following protocol: pre-denaturation at 95 °C for 3 min followed by 38 cycles (denaturation at 94 °C for 40 s, annealing at 56 °C for 30 s, and elongation at 72 °C for 45 s). Each PCR reaction was performed in 25 μL of reaction mixture, containing 1–10 ng of matrix DNA, 0.4 μM of each primer, 5x ScreenMix PCR mix with Taq polymerase (Eurogen Ltd., Russia). Amplification products were detected by electrophoresis in 1 % agarose gel, staining with ethidium bromide and visualization under UV light. PCR products were sequenced in both directions using a standard BigDye Terminator Cycle Sequencing Ready Reaction Kit on an ABI PRISM 3130 analyzer (Applied Biosystems Inc.). The obtained 28S fragments were aligned in BIOEDIT software (Hall, 1999), L. mediterraneus (JN996829, JN996828, JN996827) and L. cephali (JN996830) were used as reference sequences. All nucleotide sequences obtained in this study are deposited in the GenBank: L. mediterraneus (MZ413895– MZ413898) and L. cephali (MZ413887–MZ413893).

**Fig. 1.**
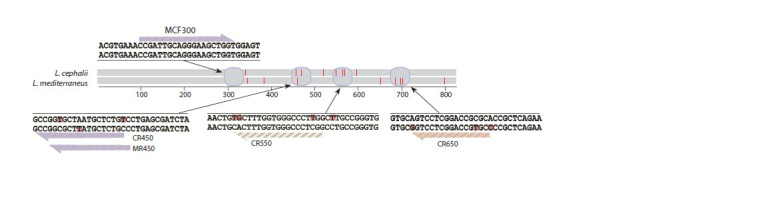
Conserved and polymorphic regions of the 28S ribosomal gene for L. mediterraneus and L. cephali

Selection of primers for genotyping L. mediterraneus and L. cephali. Variability analysis of the 28S ribosomal gene fragment showed no intraspecific variability for this genetic marker. All nucleotide sequences for each monogenean species parasitising on flathead mullet from both the Mediterranean Sea (JN996829, JN996828, JN996827, JN996830) and the Black Sea (this work) were identical. Seven sequences of the 28S fragment for L. mediterraneus and eight for L. cephali were analysed. At the same time, several sites with mutations typical for L. mediterraneus and L. cephali in the region of 450–480, 540–570 and 680–705 bp were found (Fig. 1).

DNA regions that differed by at least 3 nucleotide substitutions between the two species were selected for genotyping. Primers flanking the polymorphic regions were designed using the internet resource https://benchling.com/. All developed primers are presented in Table 1.

**Table 1.**
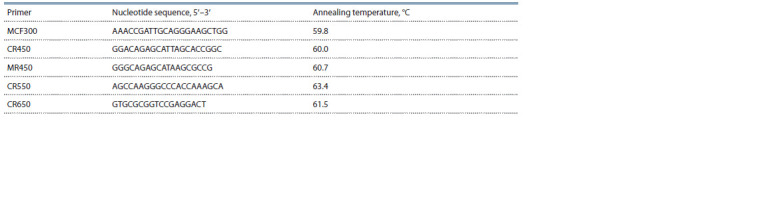
Sequences of developed primers used for genotyping

All reverse primers were tested for their level of identity to other species using the blastn algorithm against the NCBI genetic database. Only the reverse primers were tested, as they are responsible for identifying the species. The primers CR450 and CR550 showed 100 % identity with 100 % coverage only to the species L. cephali. The MR450 primer, apart from 100 % identity to L. mediterraneus, also showed the same identity to L. saladensis (GenBank number KF442629). This species occurs only off the coast of Brazil and inhabits a different host, Mugil liza. The situation with primer CR650 is similar: in addition to 100 % identity with L. cephali, there is also 100 % identity with L. heteronchus (GenBank number JN996812). This parasite also inhabits another host, Planiliza saliens. Thus, among all known flathead mullet parasites, the developed primers allow identifying two species of L. mediterraneus and L. cephali, which makes it possible to use them not only in the Azov-Black Sea basin.

Analysis of amplified fragment lengths. Two versions of the primer mixture were selected for genotyping based on the analysis of different amplicon lengths. In the first case, the amplicon lengths specific to L. mediterraneus and L. cephali differed by 120 nucleotides, and in the second, by 250 nucleotides (Table 2). The essence of the approach we developed is as follows. Three primers are added to the PCR mix instead of two primers (as in traditional PCR). One primer (forward, U178) is complementary to the conserved region of 28S and will be annealed in both species accordingly. The second primer (reverse primer) was designed for a site that differs between the two species by several mutations. In this mixture it is the MR450 primer, which is complementary to the sequence specific to the L. mediterraneus. A third primer (reverse, CR550 or CR650) was also developed for a site that differs between the two species by several mutations, but it is complementary to L. cephali.

Thus, depending on the DNA matrix, only one of the two reverse primers will be annealed and the product will be produced. The reverse primers are chosen so that the product will be 630 bp long when MR450 is annealed, but the amplicon will be longer when the other reverse primer is annealed. So, with the CR550 primer the length will be 750 bp and with the CR650 the length will be 880 bp. By performing a PCR reaction with the three primers, two Ligophorus species can be distinguished based on the length of the amplicons.

The PCR mixture and the amplification conditions were the same in both variants. The volume of the reaction mixture was 20 μL, and the final concentration of each primer (Eurogen, Russia) was 0.25 μM. The amplification was carried out according to the following protocol: pre-denaturation at 95 °C – 3 min followed by 38 cycles (denaturation at 94 °C – 40 s, annealing at 56 °C – 30 s, elongation at 72 °C – 45 s). The amplification products were detected by electrophoresis in 1 % agarose gel, staining with ethidium bromide and visualization in UV light. Monogenea species were characterized by their amplicon length, shown in Table 1.

Allele-specific end-point and real-time PCR. Genotyping based on allele-specific PCR with detection at the endpoint, as in real-time, was performed in a 20 μL reaction mixture. The final concentration of each primer (Eurogen, Russia) was 0.2 μM. The primer pairs used for each approach are listed in Table 2. Amplification with detection of PCR product at the end-point was performed according to the following protocol: pre-denaturation at 95 °C – 3 min followed by 38 cycles (denaturation at 94 °C – 40 s, annealing at 60 °C – 30 s, elongation at 72 °C – 30 s).

**Table 2.**
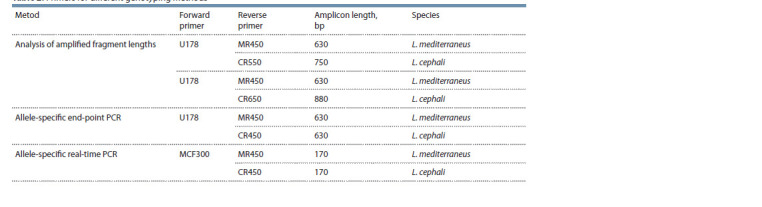
Primers for different genotyping methods

Each sample was analysed in three replicates when testing the genotyping method by allele-specific real-time PCR. The volume and composition of the reaction mixture were not changed, whereas the amplification conditions were changed: pre-denaturation at 95 °C for 3 min followed by 40 cycles (denaturation at 95 °C for 10 s, annealing at 60 °C for 10 s and synthesis at 72 °C for 30 s). In the end, a melting curve analysis was performed to evaluate the formation of primer dimers.

## Results and discussion

Morphological species identification

**Fig. 2.**
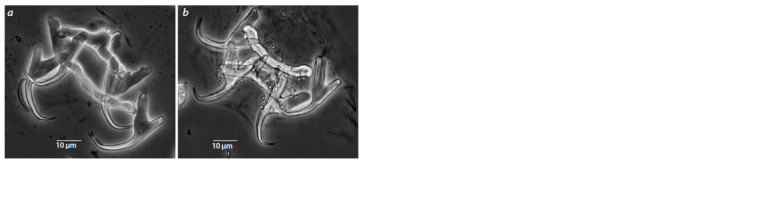
Structures of the attachment discs of L. cephali (a) and L. mediterraneus (b) collected from the gills of Mugil cephalus in the Black Sea off the coast of Crimea.

Among the 20 collected specimens, 2 species were identified by morphology (Fig. 2): 9 specimens of L. cephali, sample numbers 2, 3, 4, 6, 7, 10, 16, 17, 19, and 11 specimens of L. mediterraneus, sample numbers 1, 5, 8, 11, 12, 13, 14, 15, 18, 20, 21.

Species identification using different genotyping methods

Morphological analysis was performed for all 20 individuals, based on which the monogenic species were identified. Analysis of the nucleotide sequences of the 28S ribosomal gene fragment obtained by Sanger sequencing confirmed the morphological identification of 11 individuals and allowed us to distinguish between the two species (see Fig. 1). All 20 Ligophorus individuals were then subjected to the methods described above for separating the two species by allelespecific PCR to assess their performance.

**Fig. 3.**
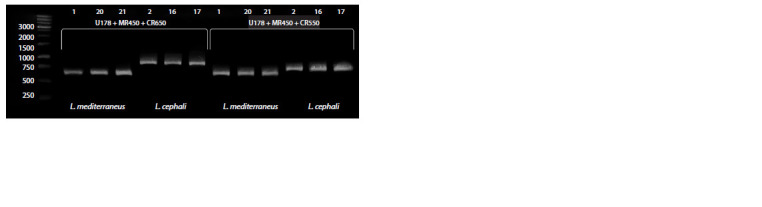
Typing of DNA samples using amplified fragment length analysis. Here and in Fig. 4: the numbers of test specimens and the primer mixture used are shown at the top, while the species that were identified based on morphology and molecular taxonomy are shown at the bottom.

The method of genotyping based on PCR product length analysis is based on using two polymorphic regions of the 28S ribosomal gene and has been described in detail above. This approach separated L. cephali and L. mediterraneus species (Fig. 3). When both primer mixture variants (U178+MR450+CR650 and U178+MR450+CR550) were used, amplification of PCR products with only one reverse primer, which had complete complementarity to the 28S region, was observed for all individuals

**Fig. 4.**
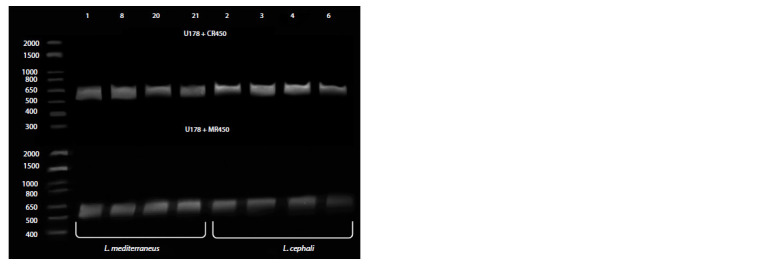
Typing of DNA samples using allele-specific PCR with end-point detection

For genotyping based on allele-specific PCR with end-point detection, two amplification reactions with different primer compositions were performed for each sample. In one version, the reverse primer was complementary to the 28S gene region characteristic of L. mediterraneus (MR450); in the other, it was complementary to the same 28S gene region specific to L. cephali (CR450). The primers differed by 3 nucleotides. Using this approach, it was not possible to select amplification conditions that would not result in annealing of primers that are not fully complementary. As a result, when detected in an agarose gel at the end-point, PCR products were always detected, although with different intensities (Fig. 4).

**Fig. 5.**
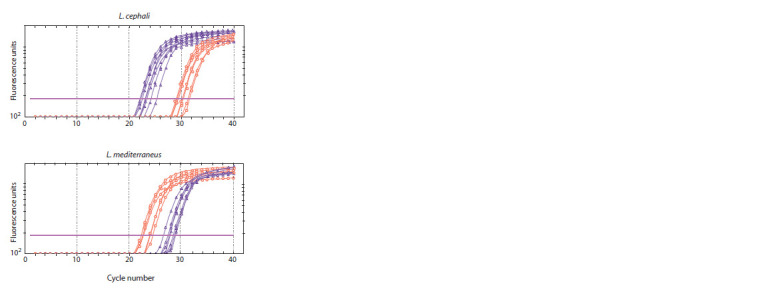
Typing of DNA samples by allele-specific real-time PCR. On the ordinate axis, the values are given in logarithmic scale. Purple indicates amplification curves for a mixture of primers MСF300+CR450, yellow for MСF300+MR450.

At the same time, using this approach, but with real-time detection, allows the two species to be distinguished (Fig. 5). It is due to the different accumulation rates of PCR products when using fully and partially complementary primers. In this approach, the direct primer has been replaced to obtain shorter amplification products, which is recommended for real-time PCR. Two amplification reactions are also performed for each individual, and then the species is determined by the lower Ct value (number of the cycle in which the fluorescence signal crosses the threshold line). Accumulation of the products is faster when the primer and the matrix of the tested DNA are entirely complementary. 100 % concordance in identification by allele-specific PCR with real-time detection with morphological analysis and sequencing data was shown for all individuals.

## Conclusion

This work developed a molecular genetic approach to rapidly distinguish between L. mediterraneus and L. cephali inhabiting Mugil cephalus in the Azov-Black Sea basin. Of the three approaches tested, two (amplified fragment length analysis method and allele-specific real-time PCR) allowed a reliable distinction between these two monogenean species. The use of allele-specific PCR with end-point detection of amplification products is inefficient because annealing and product accumulation occur for both primers complementary to L. mediterraneus and L. cephali. The approach using a PCR mixture containing three primers proposed in this work is the most cost-effective. The allele-specific real-time PCR method can be considered as the fastest and most efficient, the disadvantage of which is only its relatively high cost. Nevertheless, the developed approach is much faster and more cost-effective than sequencing the nucleotide sequences of the 28S ribosomal gene fragment.

The proposed genotyping methods can be used to rapidly separate two flatworms of the genus Ligophorus when assessing the degree of infestation of the flathead mullet with these parasites in the Azov-Black Sea region. It should also be noted that based on the data on 28S nucleotide sequences for other parasites of this genus, our developed primers have 100 % identity only with these two species of all that inhabit the flathead mullet. It allows them to be used in other parts of the world’s oceans as well. The developed approach is vital when carrying out various works to study these species, such as studying the distribution of these species, changes in the ratio of two species on one host individual, competition of these species, the influence of various factors on their abundance, etc. In addition, the results obtaineddemonstrate the promise of developing such approaches to estimate the abundance of other monogenic species, including pathogens.

## Conflict of interest

The authors declare no conflict of interest.

## References

Bakke T.A., Cable J., Harris P.D. The biology of gyrodactylid monogeneans:
the “Russian-Doll Killers”. Adv. Parasitol. 2007;64:161-376.
DOI 10.1016/S0065-308X(06)64003-7.

Blasco-Costa I., Mìguez-Lozano R., Sarabeev V., Balbuena J.A. Molecular
phylogeny of species of Ligophorus (Monogenea: Dactylogyridae)
and their affinities within the Dactylogyridae. Parasitol.
Int. 2012;61(6):619-627. DOI 10.1016/j.parint.2012.06.004.

Caltran H., Silan P., Roux M. Ligophorus imitans (Monogenea)
Ectoparasite de Liza ramada (Teleostei). I. Populations naturelles et
variabilité morphologique. Ecologie. 1995;26(2):95-104.

Cribb T.H., Chisholm L.A., Bray R.A. Diversity in the Monogenea and
Digenea: does lifestyle matter? Int. J. Parasitol. 2002;32(3):321-
328. DOI 10.1016/S0020-7519(01)00333-2.

Dmitrieva E., Dimitrov G. Variability in the taxonomic characters
of Black Sea gyrodactylids (Monogenea). Syst. Parasitol.
2002;51(3):199-206. DOI 10.1023/A:1014594614921.

Dmitrieva E.V., Gerasev P.I., Gibson D.I., Pronkina N.V., Galli P.
Descriptions of eight new species of Ligophorus Euzet &
Suriano, 1977 (Monogenea: Ancyrocephalidae) from Red Sea mullets.
Syst. Parasitol. 2012;81(3):203-237. DOI 10.1007/s11230-011-
9341-8.

Dmitrieva E.V., Gerasev P.I., Merella P., Pugachev O.N. Redescription
of Ligophorus mediterraneus Sarabeev, Balbuena & Euzet,
2005 (Monogenea: Ancyrocephalidae) with some methodological
notes. Syst. Parasitol. 2009a;73(2):95-105. DOI 10.1007/
s11230-009-9177-7.

Dmitrieva E.V., Gerasev P.I., Merella P., Pugachev O.N. Redescriptions
of Ligophorus cephali Rubtsova, Balbuena, Sarabeev, Blasco-Costa
& Euzet, 2006 and L. chabaudi Euzet & Suriano, 1977 (Monogenea:
Ancyrocephalidae), with notes on the functional morphology
of the copulatory organ. Syst. Parasitol. 2009b;73(3):175-191.
DOI 10.1007/s11230-009-9192-8.

Ergens R., Gelnar M. Experimental verification of the effect of temperature
on the size of hard parts of opisthaptor of Gyrodactylus
katharineri Malmberg, 1964 (Monogenea). Folia Parasitologica.
1985;32(4):377-380.

Fromm B., Burow S., Hahn C., Bachmann L. MicroRNA loci support
conspecificity of Gyrodactylus salaris and Gyrodactylus thymalli
(Platyhelminthes: Monogenea). Int. J. Parasitol. 2014;44(11):787-
793. DOI 10.1016/j.ijpara.2014.05.010.

Fromm B., Worren M.M., Hahn C., Hovig E., Bachmann L. Substantial
loss of conserved and gain of novel microRNA families in flatworms.
Mol. Biol. Evol. 2013;30(12):2619-2628. DOI 10.1093/
molbev/mst155.

Gusev A.V. Method for Сollecting and Processing Materials on Monogeneans
Parasitizing Fish. Leningrad: Nauka Publ., 1983. (in Russian)

Gusev A.V., Pugachev O.N., Ergens R.R., Khotenovskiy I.A. Class
Monogenea. In: Bauer O.N., Gusev A.V. (Eds.) Key to the Parasites
of Freshwater Fish of the USSR Fauna. Vol. 2. Leningrad: Nauka
Publ., 1985;10-387. (in Russian)

Hall T.A. BioEdit: a user-friendly biological sequence alignment editor
and analysis program for Windows 95/98/NT. Nucleic Acids Symp.
Ser. 41. 1999;95-98.

Kalafi E.Y., Tan W.B., Town C., Dhillon S.K. Automated identification
of Monogeneans using digital image processing and K-nearest
neighbour approaches. BMC Bioinform. 2016;17(19):511.
DOI 10.1186/s12859-016-1376-z.

Khang T.F., Soo O.Y.M., Tan W.B., Lim L.H.S. Monogenean anchor
morphometry: systematic value, phylogenetic signal, and evolution.
PeerJ. 2016;4:e1668. DOI 10.7717/peerj.1668.

Littlewood D.T.J. Platyhelminth systematics and the emergence
of new characters. Parasite. 2008;15:333-341. DOI 10.1051/
parasite/2008153333.

Littlewood D.T.J., Curini-Galletti M., Herniou E.A. The interrelationships
of Proseriata (Platyhelminthes: Seriata) tested with molecules
and morphology. Mol. Phylogenet. Evol. 2000;16(3):449-466.
DOI 10.1006/mpev.2000.0802.

Lockyer A.E., Olson P.D., Littlewood D.T.J. Utility of complete
large and small subunit rRNA genes in resolving the phylogeny
of the Neodermata (Platyhelminthes): implications and a review
of the cercomer theory. Biol. J. Linn. Soc. 2003;78(2):155-171.
DOI 10.1046/j.1095-8312.2003.00141.x.

Marchiori N.C., Pariselle A., Pereira J.J., Agnèse J.-F., Durand J.-D.,
Vanhove M.P.M. A comparative study of Ligophorus uruguayense
and L. saladensis (Monogenea: Ancyrocephalidae) from Mugil
liza (Teleostei: Mugilidae) in southern Brazil. Folia Parasitol.
2015;62:024. DOI 10.14411/fp.2015.024.

Mieszkowska A., Górniak M., Jurczak-Kurek A., Ziętara M.S. Revision
of Gyrodactylus salaris phylogeny inspired by new evidence
for Eemian crossing between lineages living on grayling in
Baltic and White sea basins. PeerJ. 2018;6:e5167. DOI 10.7717/
peerj.5167.

Mladineo I., Šegvić-Bubić T., Stanić R., Desdevises Y. Morphological
plasticity and phylogeny in a monogenean parasite transferring between
wild and reared fish populations. PLoS One. 2013;8(4):e62011.
DOI 10.1371/journal.pone.0062011.

Olstad K., Bachmann L., Bakke T.A. Phenotypic plasticity of taxonomic
and diagnostic structures in gyrodactylosis-causing flatworms
(Monogenea, Platyhelminthes). Parasitology. 2009;136:1305-1315.
DOI 10.1017/S0031182009990680.

Pakdee W., Ogawa K., Pornruseetriratn S., Thaenkham U., Yeemin
T. The first record of Ligophorus Euzet & Suriano, 1977
(Monogenea: Dactylogyridae) on Crenimugil buchanani
(Teleostei: Muglidae) from Thailand based on morphological and
molecular analyses. J. Helminthol. 2019;93(6):752-762. DOI
10.1017/S0022149X1800072X.

Poisot T., Verneau O., Desdevises Y. Morphological and molecular
evolution are not linked in Lamellodiscus (Plathyhelminthes,
Monogenea). PLoS One. 2011;6(10):e26252. DOI 10.1371/journal.
pone.0026252.

Pugachev O.N., Gerasev P.I., Gussev A.V., Ergens R., Khotenowsky I.
Guide to Monogenoidea of freshwater fish of Palaearctic and Amur
regions. Milan: Ledizione-Ledi Publ., 2009.

Rodríguez-González A., Miguez-Lozano R., Llopis-Belenguer C.,
Balbuena J.A. Phenotypic plasticity in haptoral structures of
Ligophorus cephali (Monogenea: Dactylogyridae) on the flathead
mullet (Mugil cephalus): a geometric morphometric approach.
Int. J. Parasitol. 2015;45(5):295-303. DOI 10.1016/
j.ijpara.2015.01.005.

Rubio-Godoy M. Fish host-monogenean parasite interactions, with
special reference to Polyopisthocotylea. In: Terrazas L.I. (Ed.)
Advances in the Immunobiology of Parasitic Diseases. Thiruvananthapuram:
Research Signpost, 2007;91-109.

Soo O.Y.M., Tan W.B., Lim L.H.S. Three new species of Ligophorus
Euzet & Suriano, 1977 (Monogenea: Ancyrocephalidae) from
Moolgarda buchanani (Bleeker) off Johor, Malaysia based on morphological,
morphometric and molecular data. Raffles Bull. Zool.
2015;63:49-65.

Strona G., Montano S., Seveso D., Paolo G., Fattorini S. Identification
of Monogenea made easier: a new statistical procedure for an automatic selection of diagnostic linear measurements in closely related
species. J. Zoolog. Syst. Evol. Res. 2014;52(2):95-99. DOI 10.1111/
jzs.12050.

Tokarev Yu.S., Vasilieva A.A., Grushevaya I.V., Malysh Yu.M.
Multilocus genotyping as a modern approach to the diagnosis of
microsporidia, obligate intracellular parasites of animals. Problemy
Sovremennoi Nauki i Obrazovaniya = Problems of Modern Science
and Education. 2015;12:51-55. (in Russian)

Vanhove M.P.M., Tessens B., Schoelinck C., Jondelius U., Littlewood
D.T.J., Artois T., Huyse T. Problematic barcoding in flatworms:
a case-study on monogeneans and rhabdocoels (Platyhelminthes).
ZooKeys. 2013;365:355-379. DOI 10.3897/zookeys.365.5776.

Vignon M. Putting in shape – towards a unified approach for the
taxonomic description of monogenean haptoral hard parts. Syst.
Parasitol. 2011;79(3):161-174. DOI 10.1007/s11230-011-9303-1.

Yamaguti S. Systema helminthum. Vol. IV. Monogenea and Aspidocotylea.
New York; London: Intersci. Publ., 1963.

